# INDY as a Therapeutic Target for Cardio-Metabolic Disease

**DOI:** 10.3390/metabo12030244

**Published:** 2022-03-14

**Authors:** Dominik Pesta, Jens Jordan

**Affiliations:** 1 German Aerospace Center (DLR), Institute of Aerospace Medicine, D-51147 Cologne, Germany; jens.jordan@dlr.de; 2 Center for Endocrinology, Diabetes and Preventive Medicine (CEDP), University Hospital Cologne, D-50931 Cologne, Germany; 3 Cologne Excellence Cluster on Cellular Stress Responses in Aging-Associated Diseases (CECAD), University of Cologne, D-50931 Cologne, Germany

**Keywords:** citrate transport, cardiovascular disease, metabolic disease, INDY (I’m Not Dead, Yet), SLC13A5, diabetes, insulin resistance, longevity, obesity

## Abstract

Decreased expression of the plasma membrane citrate transporter INDY (acronym I’m Not Dead, Yet) promotes longevity and protects from high-fat diet- and aging-induced metabolic derangements. Preventing citrate import into hepatocytes by different strategies can reduce hepatic triglyceride accumulation and improve hepatic insulin sensitivity, even in the absence of effects on body composition. These beneficial effects likely derive from decreased hepatic de novo fatty acid biosynthesis as a result of reduced cytoplasmic citrate levels. While in vivo and in vitro studies show that inhibition of INDY prevents intracellular lipid accumulation, body weight is not affected by organ-specific INDY inhibition. Besides these beneficial metabolic effects, INDY inhibition may also improve blood pressure control through sympathetic nervous system inhibition, partly via reduced peripheral catecholamine synthesis. These effects make INDY a promising candidate with bidirectional benefits for improving both metabolic disease and blood pressure control.

## 1. Introduction

The term cardiometabolic disease originated from the observation that metabolic and cardiovascular disease, such as arterial hypertension, often occur in the same patients, may have common underlying mechanisms, and require a comprehensive therapeutic approach. In this review we will provide an overview on contributions of citrate transport through INDY (I’m Not Dead Yet), the sodium-coupled citrate transporter SLC13A5, to cardiometabolic disease traits. Moreover, we will explore the therapeutic potential of this approach in the cardiovascular disease continuum.

### Citrate as a Central Mediator of Cellular Energy Metabolism

**Figure 1:**
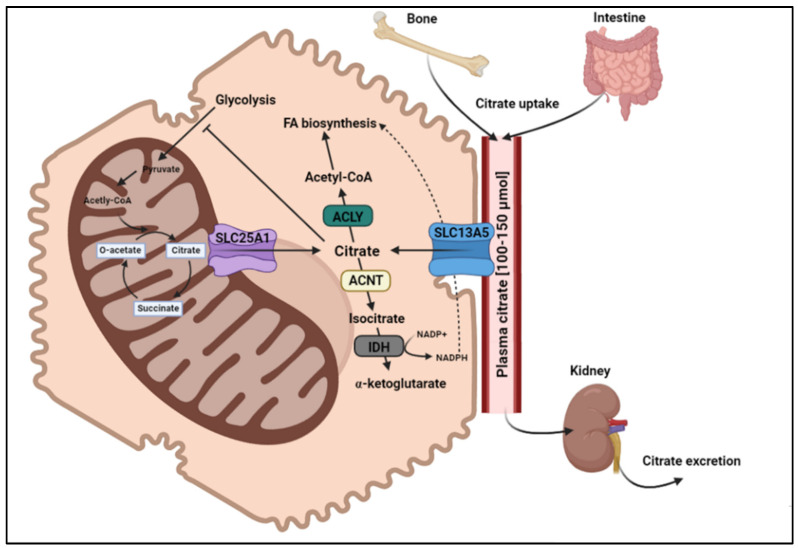
**Systemic and cellular citrate homeostasis.** Plasma citrate levels are maintained between 100–150 µmol either from intestinal absorption or from bone resorption and via urinary excretion. Citrate levels in the cytoplasm of hepatocytes are regulated by export from mitochondria via the mitochondrial citrate carrier SLC25A1 and by uptake from the circulation via the sodium-coupled citrate transporter SLC13A5. In the cytosol, citrate is a precursor for fatty acid synthesis, where the enzyme ATP-citrate lyase (ACLY) cleaves citrate to oxaloacetate and acetyl-CoA, the latter being a necessary building block for endogenous fatty acid and cholesterol biosynthesis. The NADPH required for fatty acid biosynthesis derives from the conversion of isocitrate to alpha-ketoglutarate via isocitrate dehydrogenase (IDH) after isocitrate has been produced from citrate by cytoplasmic aconitase (ACNT). Citrate also allosterically inhibits phosphofructokinase-1, the pacemaker enzyme of glycolysis, and by this means influences hepatic rates of glycolysis. Abbreviations: FA-fatty acid; O-acetate-oxaloacetate. Created with biorender.com.

The tricarboxylic acid trianion citrate (3^−^) is a key metabolite in intermediary metabolism. As a precursor to lipid and cholesterol synthesis, citrate is an important link between glucose and lipid metabolism. Citrate plays a crucial role in intermediary hepatic energy metabolism and intracellular signaling, mediating immunity and inflammation [1]. The major sources maintaining plasma citrate levels include bone resorption, intestinal absorption from dietary intake, or cellular metabolism via the tricarboxylic acid cycle (Figure 1). The citrate transport protein INDY was originally described in *Drosophila*, where its partial loss has been shown to increase lifespan [2]. In mammalians, distinct solute carrier (SLC) transporters mediate cellular uptake and subcellular transport of the tricarboxylate citrate [3]. Although there are similarities between species regarding biological function, inter-species comparisons reveal distinct transport and structural characteristics [4]. In the tricarboxylic acid cycle, citrate is oxidized to provide cellular adenosine trisphosphate (ATP) after being synthetized from acetyl-CoA and oxaloacetate. Citrate levels in the cytoplasm are regulated by mitochondrial export via the mitochondrial citrate carrier (CIC) encoded by the *SLC25A1* gene, and uptake from the circulation via the sodium-coupled citrate transporter (NaCT), also known as SLC13A5 [5], which is highly expressed in the mammalian liver, testis, and brain [6]. An overview of *SLC13A5* expression levels in various human tissues is provided by Li et al. [7]. Citrate acts as a precursor for fatty acid synthesis in the cytoplasm, where the cytosolic enzyme ATP-citrate lyase (ACLY) converts citrate to oxaloacetate and acetyl-CoA, the latter being a crucial building block for endogenous fatty acid and cholesterol biosynthesis (Figure 1) [8]. In fact, cytosolic citrate concentration has been shown to directly correlate with fatty acid synthesis rates [9,10]. High cytoplasmic citrate levels may, thus, stimulate hepatic de novo fatty acid biosynthesis and promote the development of non-alcoholic fatty liver (NAFL) [11]. As a potent allosteric regulator, citrate inhibits phosphofructokinase-1—the pacemaker enzyme of glycolysis—and stimulates fructose-1,6-bisphosphatase, an important regulatory enzyme in gluconeogenesis, thereby affecting hepatic rates of glycolysis and gluconeogenesis [12,13]. As such, metformin has lately been discovered to suppress *SLC13A5* expression [14]. Due to its role in cellular energy metabolism, manipulating cytoplasmic citrate levels in hepatocytes may be a promising therapeutic approach for metabolic disorders such as type 2 diabetes or non-alcoholic fatty liver disease (NAFLD), which are both linked to hepatic lipid accumulation and insulin resistance [15].

Along these lines, one aspect needs to be considered: while loss-of-function mutations in Drosophila or deletion of *Slc13a5* in mice may convey survival and metabolic benefits [2,6], *SLC13A5* deficiency in humans results in a recessive neurological disorder known as early infantile epileptic encephalopathy-25 (EIEE-25) [16,17]. Although *Slc13A5* null mice show some neurological abnormalities, neurological dysfunction does not seem to be present in mice to the same extent [18]. Based on these different effects of NaCT deficiency in the brain and the periphery, it has been suggested that NaCT inhibitors that do not permeate the blood-brain barrier could have advantages [19]. It may thus be prudent to avoid complete and sustained NaCT blockade when applying brain-permeable NaCT inhibitors. 

## 2. Liver-Specific Effects of INDY—From Mouse to Man?

The prevalence of NAFLD is increasing globally, and NAFLD is a risk factor for cardiovascular disease, type 2 diabetes, and certain forms of cancer [20]. NAFLD is now recognized as one of the primary causes of liver cirrhosis, and affects up to 30% of Americans [21,22]. Hepatic lipid accumulation favored by excess caloric intake and physical inactivity can lead to lipotoxicity, and has therefore been linked to the development of insulin resistance in the context of metabolic diseases such as type 2 diabetes [15,23]. The growing epidemic of obesity and diabetes and currently limited treatment options for NAFLD underline the need for novel therapeutic options. 

A number of studies reported that mammalian INDY (*mINDY)* modulation impacts liver metabolism in cellular and animal models, as well as in human beings [6,24,25]. Of note, *mINDY* transcript levels are connected to NAFLD [25]. The pregnane X receptor (PXR) is involved in regulating lipid metabolism and energy homeostasis supported by a recent study indicating that *SLC13A5* is transcriptionally regulated by PXR [26]. Induction of this transporter in human primary hepatocytes is mediated by two distal responsive elements of PXR. While rifampicin, an activator of PXR, can increase lipid accumulation, knocking down *SLC13A5* expression results in considerable reduction of lipid content in HepG2 cells [27]. The finding underlines the impact of modulating this highly inducible gene in human liver. Proof-of-concept stems from a study that tested compound 2, a selective small molecule inhibitor of NaCT activity, in human hepatocytes. Compound 2 blocked the uptake of labelled citrate, and reduced citrate incorporation into triacylglycerol [28]. Oral dosing of compound 2 reduced hepatic citrate uptake by 33% in mice, and also reduced incorporation of labelled citrate into hepatic lipids in vivo [28]. Similarly, treatment with compound 2 was associated with reductions in plasma glucose, and also reversed subsequent high-fat feeding induced glucose intolerance in mice. Triacylglycerols and diacylglycerols were reduced in the livers of animals receiving compound 2 [28]. These proof-of-concept studies further underline the therapeutic potential of NaCT inhibition. 

Whole-body *mIndy* knock-out mice were protected from development of hepatic steatosis and insulin resistance after a 6-week high-fat diet feeding [6]. Non-oxidative glucose metabolism, i.e., hepatic glycogen synthesis, was elevated in *mIndy* knockout mice. These mice also exhibited a relative reduction in whole-body fat, which may in part contribute to the observed metabolic phenotype. A subsequent study recapitulated these findings using 2′-O-methoxyethyl chimeric anti-sense oligonucleotides (ASOs) in high-fat fed rats [24]. After four weeks of ASO treatment and high-fat (60%) diet feeding, hepatic triglyceride content was significantly reduced in mIndy ASO treated rats, together with increased suppression of hepatic glucose production. Body weight was similar in control ASO and in *mIndy* ASO groups, suggesting that *mIndy* knock-down mediates improvement of hepatic steatosis and insulin sensitivity independently of body weight [24]. 

Although *mIndy* expression seems to be regulated by hormonal and nutritional cues, the regulatory mechanisms are not well established. To this end, one study utilized primary rat hepatocytes and identified a cAMP-dependent and cAMP-responsive element–binding protein (CREB)–dependent mechanism of *mIndy* regulation [29]. Induction of hepatic *mIndy* in fasted rats and high-fat-diet-streptozotocin diabetic rats identified *mIndy* as a CREB-dependent target gene of glucagon [29]. Along these lines, metformin was recently recognized to suppress *NaCT* expression in HepG2 cells, possibly via decreased phosphorylation of CREB [14]. 

In obese, insulin resistant individuals with NAFLD, *mINDY* expression correlated positively with BMI, waist circumference, body fat, and robustly with the degree of steatosis determined by histology [25]. The latter correlation remained significant even after adjusting for potential confounders such as age, sex, waist circumference, and insulin resistance. IL-6 serum levels from these patients correlated positively with hepatic *mINDY* expression in a sense that elevated plasma IL-6 in these patients predicted higher *mINDY* expression levels. Induction of *mINDY* expression after treating human hepatocytes with IL-6 and in mice after IV injection of IL-6 provides supportive evidence for these findings. In line with outcomes in patients, feeding a high-fat, high-sucrose diet to nonhuman primates for two years enhanced hepatic *mINDY* expression [25]. These data show that *mINDY* expression is regulated by nutritional cues, and depends on obesity status and metabolic health in humans and that IL-6 can increase expression of *mINDY*. 

## 3. Anti-Obesity Effects of Indy

Obesity is associated with increased morbidity and mortality, and is an established risk factor for the development of insulin resistance and type 2 diabetes [30]. In brief, excessive caloric intake in combination with physical inactivity contributes to a mismatch between lipid uptake, storage capacities in white adipose tissue and lipid utilization/oxidation/export, which leads to ectopic lipid deposition in muscle and liver [31]. Ectopic lipids can then interfere with insulin signaling pathways and contribute to insulin resistance [32]. 

At least in *D. melanogaster*, decreased *Indy* expression prevents weight gain from high-calorie food [33]. This study also shows that food calorie content is directly related to the level of *Indy* transcription. Knockdown by siRNA of the *C. elegans Indy* homolog CeNAC2 also reduced whole body fat content by ~50% [34]. A six-week period of high-fat feeding resulted in decreased body weight gain and whole-body fat accumulation in *mIndy* whole-body knock-out mice compared to wildtype controls [6]. Further analyses showed an increased energy expenditure in these animals, which likely explains the reduced whole-body fat content. Mechanistically, *mIndy* knock-out decreases hepatic ATP content and ATP/ADP ratio which activates 5′ AMP-activated protein kinase, and leads to increased hepatic mitochondrial function and lipid oxidation capacity, concomitant with reduced *de novo* lipogenesis in primary *mIndy*^−/−^ hepatocytes [6]. 

Interestingly, liver-selective siRNA knockdown of *mIndy* for eight weeks in C57BL/6J mice fed a Western diet did not differently affect body weight, whole-body fat accumulation or lean mass compared to animals treated with unspecific control siRNA [35]. Respiratory exchange ratio as a crude measure of metabolic flexibility was also not different between groups. Unsurprisingly, caloric intake and energy expenditure did not differ between groups. Of note, with this knock-down approach, 35% of mIndy activity still remained [35]. Another study investigated the metabolic effects of a selective inducible hepatic *mIndy* knockdown in rats on a 60% high-fat diet [24]. Although body composition was not assessed in this study, body weight was not different between rats receiving ASO targeted against *mIndy* and the control group. Compared to constitutive knock-out models, body weight and composition seem not to be affected in models of conditional knock-down. The finding can have several reasons, including too short an experimental timeframe to induce changes in body weight, or remaining mIndy activity in the latter models as compared to genetic knock-out, which results in complete deletion of *mIndy* during the entire course of life, including prenatal development. 

Human hepatic *SLC13A5* expression is positively associated with measures of obesity, body fat and liver fat content assessed from histology [25]. Lithium treatment in humans can result in dyslipidemia and body weight gain [36]. Lithium also stimulates mINDY activity, implying a possible clinically relevant connection between increased mINDY activity and obesity in humans [37]. 

Taken together, genetic knock-out approaches seem to impact body weight and body composition while the more clinically relevant knock-down approaches do not. As potential therapeutic approaches would likely involve selective and potent inhibition of organ-specific citrate transport, it is possible that these approaches could have a limited effect on body weight or fat content. Nevertheless, indirect effects on systemic lipid metabolism could stem from reduction in hepatic lipid synthesis and subsequent decreased VLDL-export and peripheral uptake. 

## 4. Indy Contributions to Blood Pressure Control

The sympathetic nervous system has a crucial role in blood pressure regulation and promotes arterial hypertension. Chronically increased energy balance leading to adiposity and ageing is associated with increases in sympathetic activity in animal models and human beings [38,39,40]. Conversely, fasting or chronic weight loss attenuate sympathetic activity [41,42]. Reduced mIndy activity promotes a phenotype characterized by improved body composition, reduced adiposity in the face of increased caloric supply, and metabolic reprogramming akin to fasting [6]. Through these mechanisms, mIndy could regulate the sympathetic nervous system and blood pressure.

**Figure 2:**
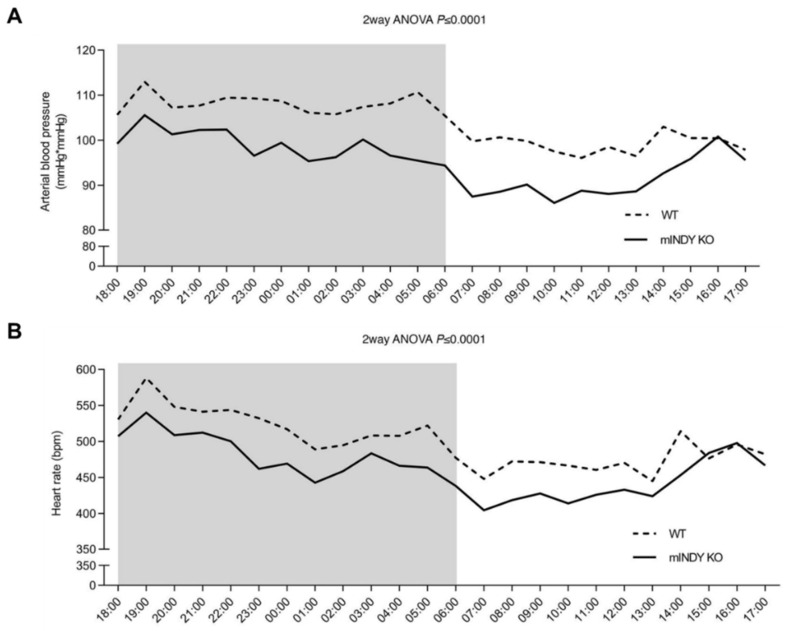
Arterial blood pressure (**A**) and heart rate (**B**) in *mIndy*-KO mice (n = 6) and WT littermate controls (n = 8) on a regular chow diet. Arterial blood pressure (**A**) monitored by a radiotelemetry system was on average 8 mmHg lower in *mIndy* knockout mice compared to WT controls. Heart rate (**B**) was on average 37 bpm lower in *mIndy* knockout mice compared with WT controls. KO-knock-out; WT-wild-type; bpm-beats per minute; see [43] for more details.

In a recent study, blood pressure was measured through implanted telemetry probes in freely moving *mIndy* knockout mice and in wildtype controls [43]. Animals were on standard chow, and body composition did not differ between groups. To spare carotid baroreceptors, which regulate sympathetic activity and blood pressure, the arterial catheter was inserted through the femoral artery. Mean blood pressure averaged over three days was 8 mmHg lower in *mIndy* knockout mice (Figure 2A). Moreover, heart rate was 37 bpm lower in *mIndy* knockout mice compared with wildtype controls (Figure 2B). The difference in blood pressure and heart rate between groups was not explained by differences in physical activity. Concomitant reductions in blood pressure and in heart rate could point towards centrally mediated sympathetic inhibition in *mIndy* knockout animals. Indeed, urinary norepinephrine and epinephrine excretion was substantially reduced in *mIndy* knockout mice [43]. 

Pharmacological ganglionic blockade interrupts parasympathetic and sympathetic efferent nerve traffic at the level of autonomic ganglia, and can be utilized to gauge sympathetic support of blood pressure [40,44,45]. Compared with wildtype controls, blood pressure and heart rate reductions with ganglionic blockade were attenuated in *mIndy* knockout mice [43]. This finding confirms the idea that reduced mIndy activity lowers blood pressure through sympathetic inhibition. Changes in sympathetic activity are usually associated with altered dynamic influences of sympathetic and parasympathetic activity on blood pressure and heart rate, which can be captured through heart rate and blood pressure variability and spontaneous baroreflex sensitivity measurements [46]. Strikingly, *mIndy* knockout mice showed an approximately 50% reduction in systolic blood pressure variability in the low-frequency range [43], which relates to sympathetic activity in mice and in human beings [38,47]. Sympathetic and parasympathetic cardiovascular activity are often regulated in a reciprocal fashion, such that reductions in sympathetic activity are associated with increases in cardiac parasympathetic activity [48]. In fact, *mIndy* knockout mice exhibited an increase in spontaneous baroreflex sensitivity, which is strongly affected by parasympathetic heart rate control [43]. 

The sympathetic inhibition in *mIndy* knockout mice may not solely be explained by central nervous mechanisms. Gene expression analysis in adrenal medullary samples from *mIndy* knockout mice showed decreased expression of catecholamine synthesis pathways. In particular, the rate limiting enzyme in catecholamine biosynthesis tyrosine hydroxylase was downregulated. In subsequent cellular experiments in a pheochromocytoma cell line, pharmacological *mIndy* inhibition decreased cellular citrate uptake and lowered norepinephrine precursor as well as native norepinephrine concentrations [43].

Overall, reduced mIndy activity appears to lower blood pressure through sympathetic nervous system inhibition. Remarkably, reduced peripheral catecholamine synthesis may contribute to the response, thus, providing an interesting target for the treatment of arterial hypertension. Indeed, observations in exceedingly rare patients with dopamine-beta-hydroxylase deficiency, the enzyme required to synthesize norepinephrine from dopamine, support the notion that peripheral catecholamine metabolism is crucial for blood pressure control [49]. Since mIndy affects weight gain in mice on high-fat diet and weight gain in turn activates the sympathetic nervous system through the leptin-melanocortin pathway [50,51], beneficial effects of mIndy on blood pressure may be greater in the presence of concomitant obesity and associated metabolic diseases. However, this idea has not been tested.

## 5. Rationale for Indy Inhibition in Patients with Cardiometabolic Disease

A potential advantage of *mINDY* inhibition in the treatment of cardiometabolic disease is that metabolic and cardiovascular traits are targeted in parallel. Patients with obesity, type 2 diabetes mellitus, NAFLD, or non-alcoholic steatohepatitis (NASH) are at increased risk for arterial hypertension, and vice versa. Concomitant metabolic disease and arterial hypertension may exacerbate cardiovascular and renal disease risk, which complicates clinical management. For example, patients with arterial hypertension and obesity require more antihypertensive medications and are, nevertheless, less likely to have their blood pressure controlled compared with patients who are hypertensive but normal weight [52,53]. Concomitant metabolic disease and arterial hypertension may also create a therapeutic dilemma when prescribing medications. Beta-blockers, which are commonly prescribed to patients with arterial hypertension, may promote weight gain [54]. Moreover, beta-blockers without vasodilating properties tend to worsen insulin sensitivity [55]. Conversely, medications that have been developed to treat metabolic disease, such as the serotonin and norepinephrine uptake inhibitor sibutramine, may worsen blood pressure control and adversely affect cardiovascular risk [56]. Thus, therapeutic strategies that address metabolic disease and at the same time improve blood pressure control may be particularly beneficial. Recent large-scale cardiovascular outcomes trials suggest that this idea is not completely off the mark. Sodium-glucose cotransporter 2 inhibitors and glucagon-like peptide 1 receptor agonists, which, in addition to improving glycemic control and body weight, also lower blood pressure, improved hard cardiovascular endpoints [57,58]. Combined angiotensin subtype 1 receptor and neprilysin inhibition, which not only lowers blood pressure, but also ameliorates insulin sensitivity [59], had a beneficial effect on cardiovascular outcomes in patients with heart failure [60]. Overall, these findings provide an impetus investigating the potential of mINDY inhibition in improving metabolic disease and blood pressure in more detail. 

## 6. Conclusions

Arterial hypertension is often accompanied by metabolic diseases such as obesity, type 2 diabetes, NAFLD, and NASH, and vice versa. Coexistence of these diseases increases the risk for further cardiovascular and renal comorbidities and complicates treatment. Although mINDY is an interesting candidate for improving these conditions, inhibition of *mINDY* would likely need to be specifically targeted to individual organs, such as the liver, in order to avoid off-target effects in the brain. While there are no genetic association studies linking SLC13A5 variants to human metabolic diseases [61], data from cell-based, mouse, and human studies indicate that inhibition of *mINDY* comprises an interesting and promising strategy to target metabolic and cardiovascular traits within the cardiometabolic disease spectrum.
